# Optimising TNM Staging of Patients with Prostate Cancer Using WB-MRI

**DOI:** 10.5334/jbr-btr.1209

**Published:** 2016-11-19

**Authors:** Vassiliki Pasoglou, Nicolas Michoux, Bertrand Tombal, Frédéric Lecouvet

**Affiliations:** 1 UCL- Saint Luc, BE

**Keywords:** Prostate Cancer, Whole Body MRI, Diffusion Weighted Imaging, TNM Staging, Three Dimensional, Bone Metastasis, Node Metastasis

## Abstract

Multiparametric Magnetic Resonance Imaging (mp-MRI) is the current standard of reference for the local staging of prostate cancer (PCa). On the other hand, despite the low sensitivity and specificity of Technetium Bone Scanning (BS) for the detection of bone metastases (BM) and of Body Computed Tomography CT for the detection of lymph node metastases (LNM), these techniques are routinely used, in the current clinical practice. Nevertheless, whole Body MRI (WB-MRI) and Positron Emission Tomography Computed Tomography (PET-CT) are emerging as robust tools for the staging of oncologic patients, including those with (PCa).

The available techniques (BS, WB-MRI, PET, CT) for the detection of BM in oncologic patients were compared and showed striking center differences in terms of anatomic sequences and planes used. This heterogeneity and the long acquisition time of WB-MRI protocols – due to the addition of multiple anatomic sequences in different planes – questioned whether a single three dimensional (3D) sequence could replace the multiple anatomic sequences used for node and bone staging of PCa. We demonstrated that WB-MRI is a credible tool for the detection of bone and node metastasis.

The second question addressed the possibility to obtain a complete TNM staging of PCa in a single MRI session. A WB-MRI protocol was developed to enable complete, T (local), N (regional) and M (distant) staging of PCa in a single session, in less than an hour. This ‘all-in-one’ protocol proved to be as efficient as the sum of exams currently in use for the staging of PCa (ie: mp-MRI of the prostate for ‘T’ staging, Thoraco-abdominal CT for ‘N’ staging and bone scintigraphy for ‘M’ staging).

## Prostate Cancer

Prostate cancer (PCa) is the most common non-skin cancer in America [1]. According to the American Cancer Society, PCa will affect one in five American men. In the United States more than two million men are living with PCa. Asian countries, especially China, have the lowest rates, while the USA, Scandinavia and Western Europe have the highest [2]. In addition, the relative incidence of PCa in African American men is higher than in Caucasians (1.6 times higher in the USA).

Men with first-degree relatives with PCa have an increased risk for developing the disease [3]. Furthermore, an X-linked or recessive model of inheritance has been suggested due to the excess risk of PCa in men with affected brothers compared to those with affected fathers [4].

The majority of men with PCa will remain asymptomatic. The symptoms – if they appear – are caused by the local extension of the disease and include dysuria, haematuria and erectile dysfunction. Sometimes the disease is detected only after a complication has occurred due to the metastatic spread, such as pathological fracture.

**Figure 1:**
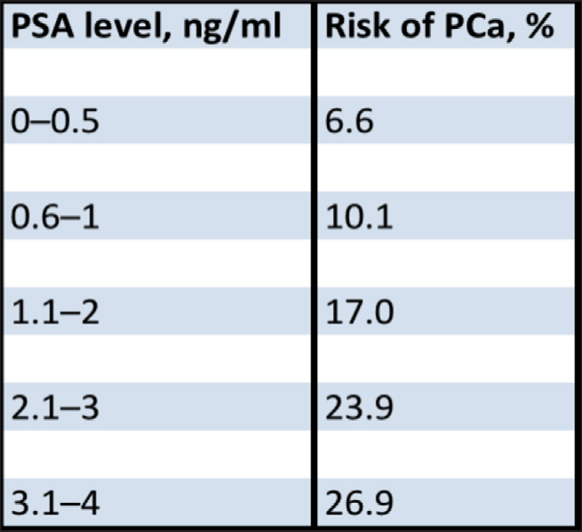
Risk of PCa in patients with low PSA levels according to Thompson et al. [5].

Two tests are currently widely used for the screening of PCa: the digital rectal examination (DRE) and the blood Prostate Specific Antigen (PSA) levels testing. The increase of PSA levels can be associated with benign conditions, such as prostatitis and prostate hyperplasia. The risk of PCa for PSA values <0.5 ng/ml is 6.6% and it increases to 26.9% for PSA values between 3.1 and 4.0 ng/ml [5] (Figure 1). It has been demonstrated that PSA-based screening reduces the rate of PCa-related deaths by 20%, even though it is associated with a high rate of over-diagnosis [6].

When the clinician suspects the presence of PCa after a DRE, a transrectal ultrasound (TRUS) with biopsies should be performed.

**Figure 2:**
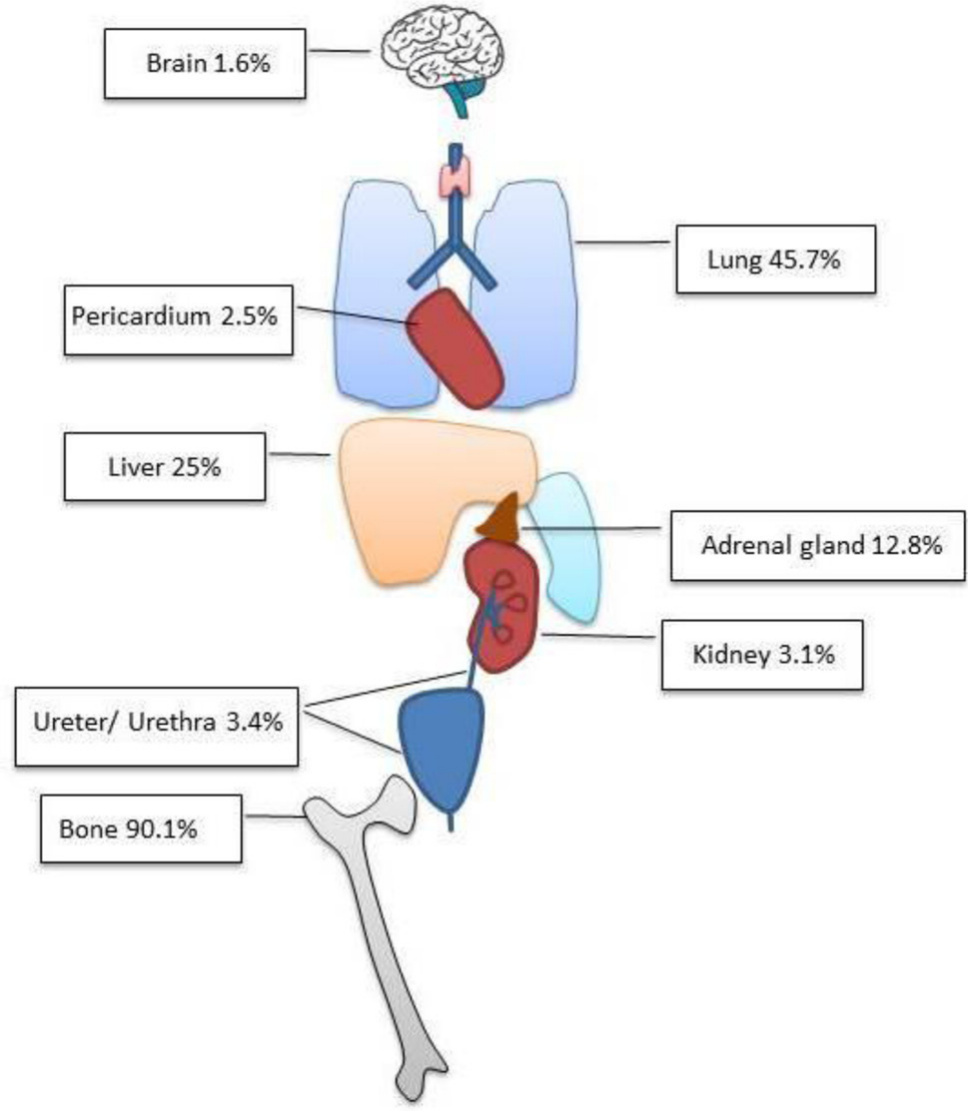
Distribution of haematogenous metastasis according to Bubendorf et al. (n = 556 patients) [7].

The most frequent sites of metastasis in patients with PCa are lymph nodes, bone, lung and liver. Bubendorf et al., carried out 19,316 autopsies in men older than 40 years old and PCa was found in 8.2% of them [7]. Lymphatic or haematogenous metastases were observed in 39.7% of all patients, and in 65.8% of patients with clinically known cancer. From the patients with distant metastasis, 90.1% of patients had bone involvement, 45.7% had lung and 25% liver metastasis (Figure 2).

The most widely used staging system for PCa is the Tumor Node Metastasis (TNM) system. For PCa, T1 refers to organ-confined tumors that are clinically and radiologically occult. T2 refers to organ-confined tumors that are clinically or radiologically apparent. T3 refers to tumors that extend outside the prostatic capsule in the form of extracapsular extension or seminal vesicle invasion. T4 refers to tumors that invade the adjacent structures. Lastly, N1 indicates the presence of locoregional LNM and M1 indicates the presence of distant metastases.

For the T staging of PCa, mp-MRI is a very effective diagnostic tool and considered the gold standard [8].

For the N staging, most of the teams use a thoraco-abdominal CT, rarely MRI. The most widely used CT and MRI criteria to determine whether a node is benign or malignant are the size, the shape, the contour and the number of lymph nodes, though their diagnostic values are debatable. For instance, according to McLoud et al., the smaller the nodal diameter criterion used to separate malignant from benign nodes, the higher the sensitivity and the lower the specificity are [9]. It has been demonstrated, however, that 10–20% of normal-sized locoregional nodes will contain tumor deposits and 30% of enlarged lymph node are benign [9,10,11]. A meta-analysis by Hövels et al., reported that both CT and MRI perform equally poorly in the detection of LNM from PCa [12]. Normal structures and other pathologic processes can mimic nodal disease. Common pitfalls include small bowel loops in close proximity to the retroperitoneum, normal ovaries, aberrant vessels (especially on non-contrast-enhanced CT), and normal anatomic variants such as a left-sided inferior vena (IVC) cava or duplicated IVC. Finally peritoneal nodules can mimic mesenteric or pelvic LNs and lymphoceles.

As the most common distant metastatic site of PCa is the skeleton which represents the initial metastatic site in more than 80% of PCa patients, most centers usually use bone scintigraphy with targeted X-rays when necessary for the M staging of the patients. The radionuclide used is Tc 99m bound to a bisphosphonate. BS has played a crucial role in tumor staging for years, but its reliability has been repeatedly questioned, mainly because of its lack of sensitivity and specificity [13,14]. Due to the high incidence of false positive results (osteoarthritis, Paget’s disease, fractures, etc.), a positive BS will – most of the times – lead to another modality (X-Ray, CT, MRI), in order to clarify the diagnosis, which leads to additional costs, waiting times and irradiation of the patient.

MRI plays a crucial role in the detection, characterization and follow-up of BMs. It possesses the unique advantage of directly exploring the bone marrow and as a result allows early detection of bone involvement. Numerous studies have demonstrated that MRI of the axial skeleton and, later, WB-MRI are superior to the current multistep BS –/+ targeted X-rays staging protocol [15,16,17,18,19,20].

**Figure 3:**
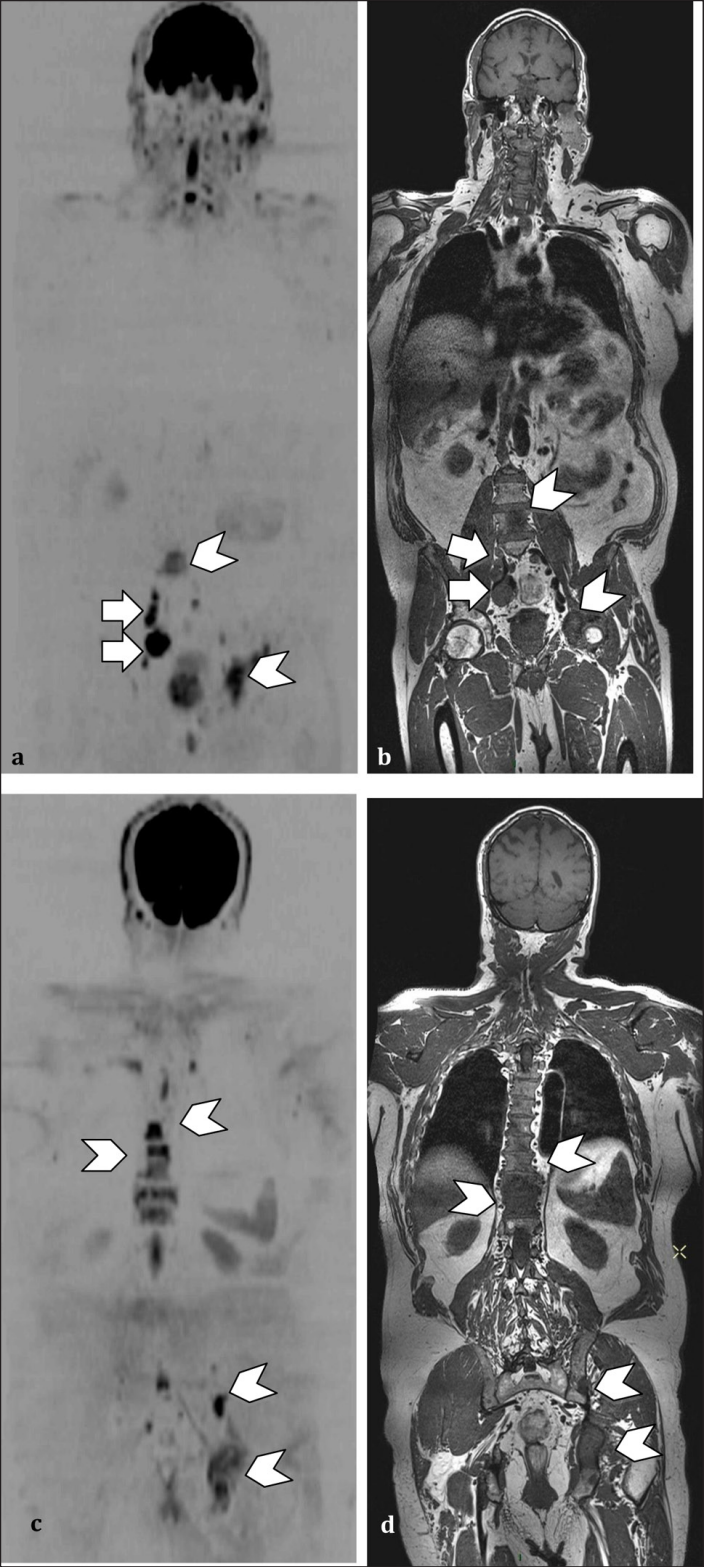
**(a and c)** Whole body DWI **(b and c)** Anatomic T1 sequence. PCa patient with nodes (arrows) and bone metastasis (arrowheads) detected by DWI and confirmed by the anatomic sequences.

The usual WB-MRI protocols include:

– T1-weighted spin echo sequences for an optimal study of the bone marrow
– Fat suppression techniques for their sensitivity concerning the detection of bone lesions
– Diffusion Weighted Imaging (DWI). The complex process of water molecule diffusion is the basis of DWI. The unrestricted motion of water molecules is called ‘free diffusion’. In biologic tissues, the movement of water is restricted, as its motion is limited by interaction with cell membranes and macromolecules. The tissue cellularity is correlated to the degree of restriction to water diffusion. In tissues with high cellularity (such as tumors), the movement of water molecules is impeded. In contrast, in regions with low cellularity or where the membranes are damaged, the motion of the water molecules is less restricted.
  The use of DWI provides functional information and enables an “at-a-glance” assessment; this reduces interpretation times of examination by driving the attention of the radiologist to abnormalities, which can then be evaluated on anatomic [T1 or Short Tau inversion recovery (STIR)] whole-body MR images [21]. Whole-body DWI is used as a supplement to anatomic whole-body MR imaging, leading to improved reader performance. Most importantly it enables concomitant bone and node staging (Figure 3) and thus it allows a global assessment of the cancer spread (N and M) in one step.

## Whole body MRI

The first studies utilizing WB-MRI were published between 1997 and 2001 [22,23,24]. WB-MRI is increasingly used for an ample variety of applications. MRI is a highly sensitive method for early detection of BM as it allows the identification of malignant cells in the bone marrow before bone remodelling [25,26,27]. It represents a convenient and cost-effective method for screening patients with malignancies [26]. Eustace et al., compared whole body STIR with Tc 99m BS in 49 patients with suspected skeletal metastases and demonstrated that WB-MRI has a better sensitivity than Tc 99m BS [28].

The main disadvantages of WB-MRI are its relatively long scanning times, motion artefacts (requiring patient cooperation or general anaesthesia) and limited specificity. However, advances in hardware and imaging techniques, including additional sequences, are reducing the impact of some of these challenges. Most importantly, the introduction of DWI radically improves the diagnostic effectiveness of WB-MRI and promotes the technique as a tool of choice for PCa staging.

## Limitations of current staging methods

Accurate distant and local staging of PCa patients is crucial in order to adapt treatment to the actual stage of the disease. Patients with bone metastasis cannot receive local treatment (surgery or radiation therapy), hence accurate detection of bone disease is essential. The development of clinical trials evaluating novel compounds and local treatment in the so-called oligo-metastatic patients has rendered the accurate evaluation of the metastatic burden indispensable [29,30,31].

For N and M staging CT and/or abdominal MRI and Tc 99m BS with targeted X-rays (if necessary) are the most widely proposed staging methods [32,33,34].

Additionally to its poor sensitivity and specificity, discussed above, the aforementioned multimodality algorithm necessitates multiple hospital visits and appointments in different departments (radiology and nuclear medicine), leading to additional times and discomfort for this group of patients (who are mostly elderly with multiple comorbidities). The delays for the interpretation of all these examinations also have to be considered.

Multimodality algorithms would advantageously be replaced by one step modalities (WB-MRI and PET) protocols which have suggested their superiority and allow the rapid assessment of total tumor and all organ evaluation [35].

Concerning PET-CT, the most commonly used and widely available radiotracer is ^18^F-FDG that is a non-specific tumor-seeking radiopharmaceutical. The use of ^18^F-FDG is based on the heightened glycolysis in tumor cells, which provokes a high uptake of ^18^F-FDG by the tumor. Unfortunately PCa cells have low glycolysis levels and as a result they are not ^18^F-FDG-avid. This fact renders FDG PET modality poorly efficient for the detection of PCa metastasis [36]. Furthermore, kidneys secrete ^18^F-FDG and as a result there is a physiological uptake by the urinary tract. This explains a suboptimal analysis of the urinary tract in general. Recently, promising Choline and Prostate-specific membrane antigen (PSMA) tracers have been developed for nuclear medicine techniques. PSMA is a cell surface protein overexpressed by PCa cells. Ligands of PSMA are labelled with ^68^Ga, Tc 99m and ^123/124/131^I for the detection of PCa metastases or local relapse [37,38,39]. Afshar-Oromieh et al. demonstrated that suspicious PCa lesions present excellent contrast as early as 1 h post-injection of PSMA [40]. In a retrospective analysis of 319 patients who underwent Ga-PSMA-ligand PET-CT, the same team found that PSMA is highly specific for PCa and detects the disease in a high percentage of patients (82.8%) [41] .

All of the methods mentioned above are irradiating and require the injection of contrast material or radioactive tracers.

Regarding WB-MRI, even though various studies demonstrated its superiority compared to the current staging methods, there is an important heterogeneity between different countries, centers, or even teams, regarding the anatomic sequences, acquisition planes and duration of the examination [42]. Thus, there is an urgent need for precision and standardization of the protocols, depending on the cancer type and the patient.

### Value of imaging in the detection of bone metastasis

We effectuated a large-scale review in order to determine the value of the different techniques for the detection of bone metastasis. We quickly observed an important heterogeneity regarding the MRI protocols used by different teams concerning WB-MRI. Our results confirmed that the recommendations for bone staging differ depending on the primary cancer.

WB-MRI was superior to BS for BM detection, independently of the primary cancer.

The addition of DWI to WB-MRI protocols significantly improves the sensitivity but the need of anatomic sequences is indisputable.

The diagnostic accuracy of WB-MRI and PET-CT appeared roughly equivalent but WB-MRI is superior in lesion-based analysis when PET-CT does better than WB-MRI in patient-based analysis, especially in breast and neuroendocrine tumors.

It has to be highlighted that an advantage of MR imaging is the lack of radiation exposure, contrast injection and its sensitivity to bone marrow infiltration regardless of the primary cancer, as PET-CT relies on the affinity of the cancer for a given tracer.

We observed a lack of studies comparing WB-MRI to PET-CT with new radiotracers, such as ^18^NaF, ^11^C and ^18^F Choline and labelled PSMA.

In general, cancer staging cannot be based on one technique, but it will most likely be shared between WB-MRI and PET according to the primary cancer.

### 3D T1-weighted MRI protocol for prostate cancer staging

**Figure 4:**
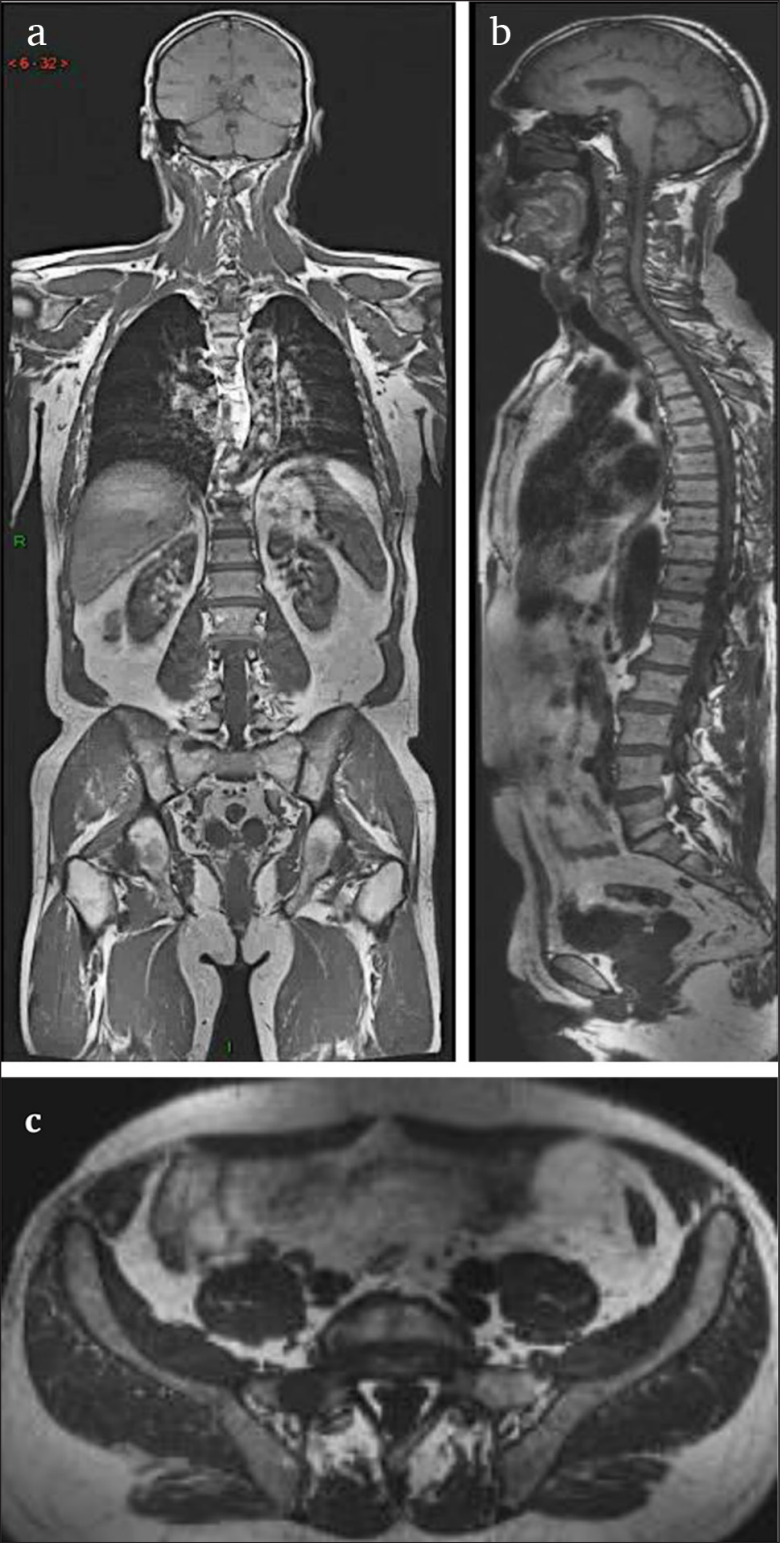
3D T1 anatomic MRI sequence reconstructed in coronal **(a)**, sagittal **(b)** and axial plane **(c)**.

Anatomic and functional DWI sequence must be acquired for the N and M staging of oncologic patients. According to our review, there is an important heterogeneity concerning the sequences and the anatomic planes that are used. The heterogeneity between teams concerns the ‘anatomic part’ of the protocol. A need for harmonisation arises, in order to standardize the WB-MRI protocols. This wide variability of sequences and planes led us to the development of a three dimensional (3D) WB-MRI sequence which can be reconstructed in any plane and could potentially stand alone as the anatomic counterpart of a WB-MRI protocol (Figure 4). We tried to simplify the WB-MRI protocol by developing a fast and reliable 3D anatomic MRI sequence for all organ (N+M) screening. We compared this new sequence with the MRI protocol routinely used in our center, which consists of a two dimensional (2D) coronal T1 WB-MRI and a sagittal proton density fat suppression sequence of the whole spine (PDFS), for concurrent bone and node staging.

The new sequence we developed is a 3D turbo spin-echo SPACE (sampling perfection with application optimized contrast using different flip angle evolutions) pulse sequence in coronal plane that was implemented to get high spatial resolution and high contrast resolution in T1, while limiting both the specific absorption rate and the acquisition time. An elliptic k-space filter was applied. Then, in-plane spatial resolution was chosen so as to be the closest to that of the 2D pulse sequence and to be ‘as isotropic as possible’. The acquisition time is 18.5 minutes.

#### Technical note

The long acquisition and post-processing times until recently limited the use of 3D sequences; however, the development of more effective imaging techniques and high-performance MR imaging workstations have allowed overcoming these problems. The reduction of the duration of the sequence is crucial. Increasing the slices’ thickness from 1 to 1.5mm can reduce the time of the acquisition but with subsequent loss in isotropy and quality of image. Another way to reduce the duration of the sequence is to increase the parallel imaging (PI) acceleration factor (we already operate in parallel imaging with an acceleration factor 3). Most modern MR systems are built in a parallel architecture, consisting of a body transmit coil and set of local receiver coils that feed into parallel channels for signal amplification and processing. In PI, information about coil positions and sensitivities can be used to reduce the number of phase-encoding steps and speed up imaging. This is quantified by the PI acceleration factor (typically between 2 and 6). The temporal and spatial resolution requirements – the field strength at which the study is performed and the object of interest – all lead to the decision to use PI acceleration.

The major advantages of PI:

– Significant reduction of image acquisition time. This is inversely related to the acceleration factor (R). If R = 2 then image acquisition time is cut in half.
– Reduction in susceptibility artefacts. The PI acquisition and reconstruction process lessens phase-related distortions in the MR signal. This is especially advantageous in echo-planar sequences.

#### Study group

Thirty consecutive patients with PCa who were considered as high risk for metastases (PSA > 20 ng/ml, Gleason ≥ 8, or T stage ≥ 3a) [34] were prospectively enrolled between February and December 2012. The mean patient age (±standard deviation) was 69 ± 3.3 years. The mean prostate-specific antigen level was 31 ± 28 ng/mL. Three patients were not enrolled because of contraindications to MR imaging.

## Results

**Figure 5:**
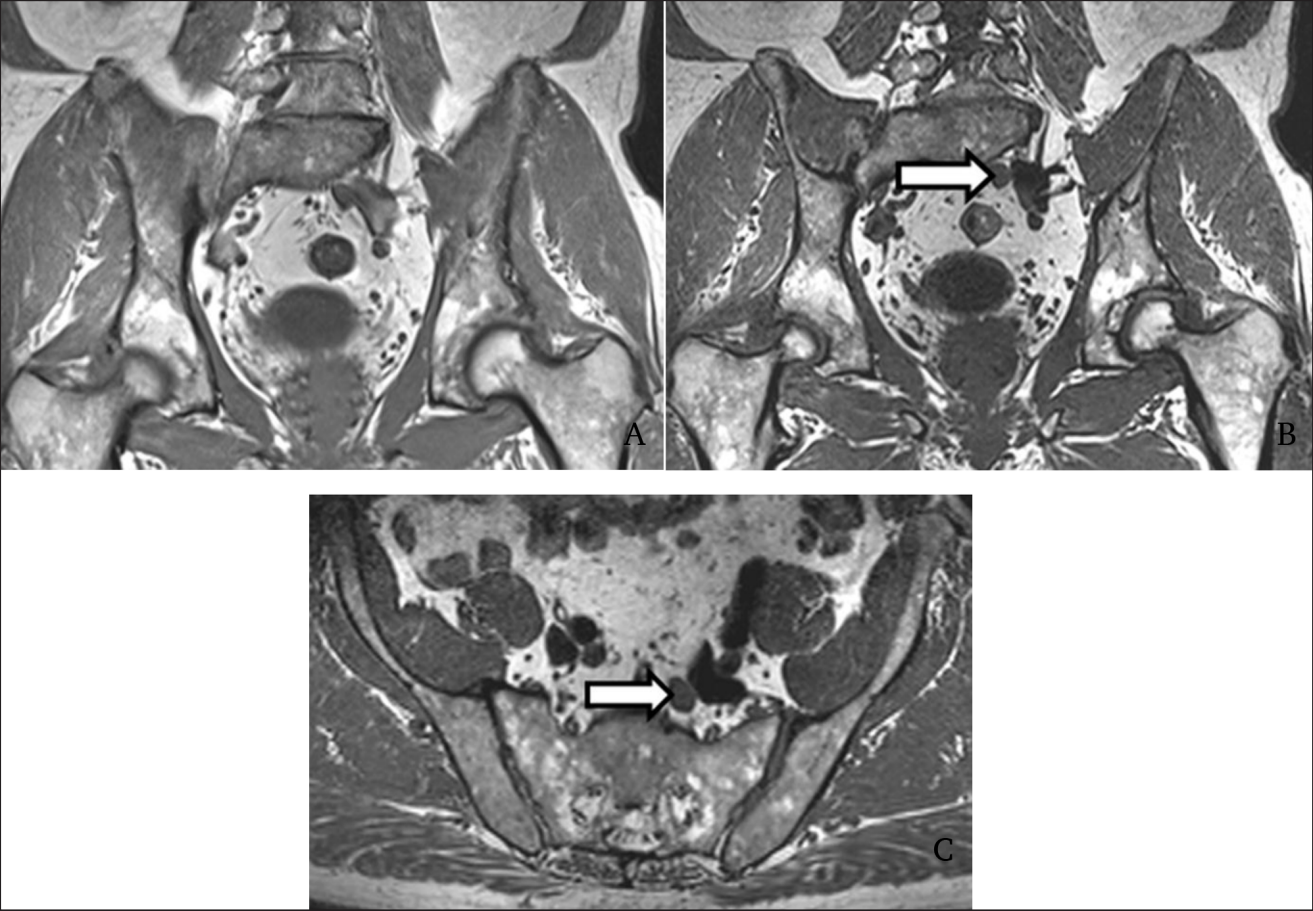
Detection of a node in a 57-year-old PCa patient. The enlarged LN was missed by both readers on 2DT1 sequence **(A)** but correctly identified by both on 3D T1 sequence **(B)**. Axial reconstruction obtained with multiplanar reformation at same level confirms the presence of an enlarged LN **(C)**. Reprinted from ‘Whole-body 3D T1-weighted MR imaging in patients with prostate cancer: feasibility and evaluation in screening for metastatic disease’ Pasoglou V., Michoux N., Peeters F., Larbi A., Tombal B. Omoumi P., Vande Berg B., Lecouvet F.E, Radiology. 2015 Apr; 275(1): 155–66. [43] With the permission of RSNA.

**Figure 6:**
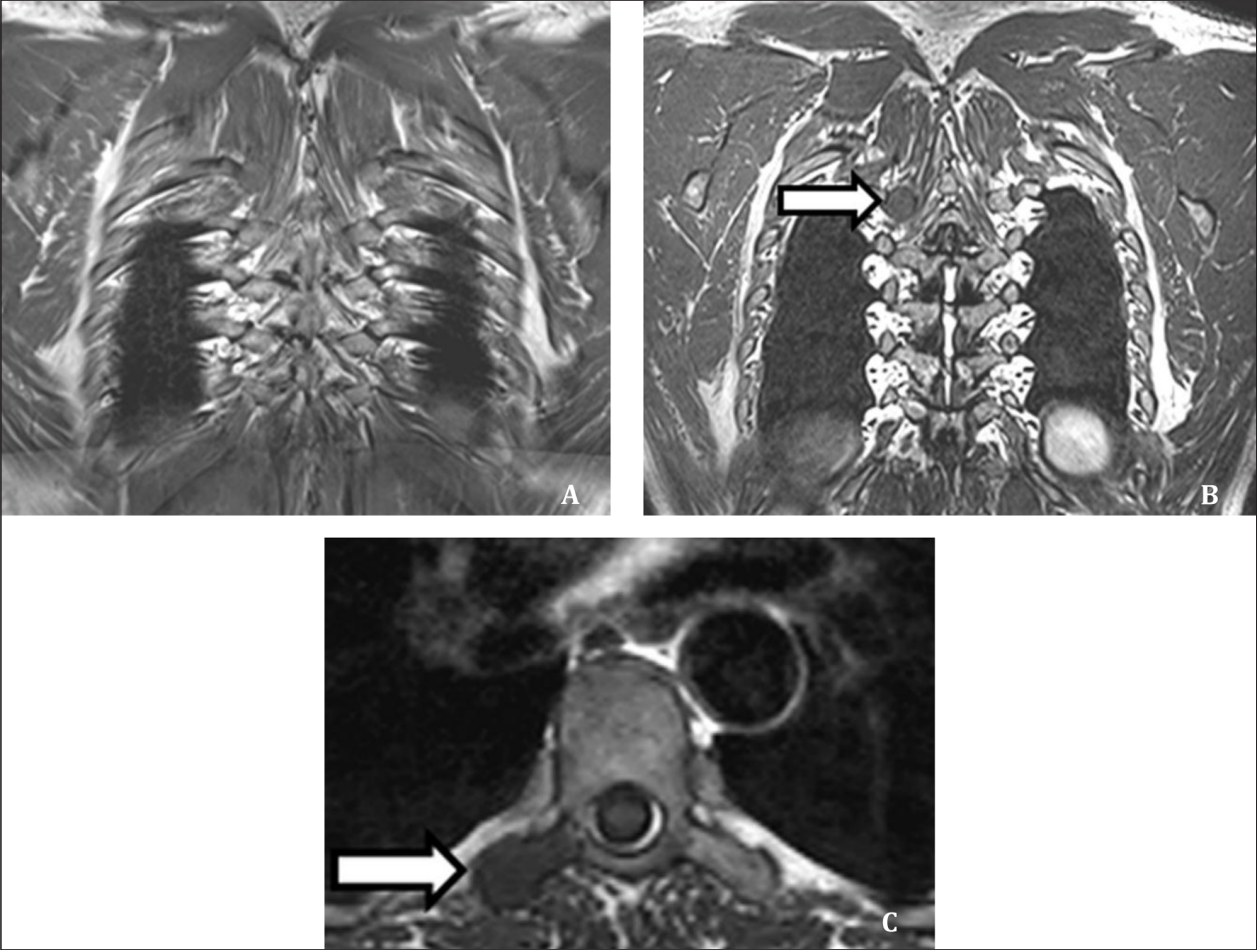
Detection of a BM in a 63 year old PCa patient. The lesion was missed by both readers on 2DT1 sequence **(A)** but correctly identified by both on 3D T1 sequence **(B)**. Axial reconstruction obtained with multiplanar reformation at same level confirms the presence of a BM **(C)**. Reprinted from ‘Whole-body 3D T1-weighted MR imaging in patients with prostate cancer: feasibility and evaluation in screening for metastatic disease’ Pasoglou V., Michoux N., Peeters F., Larbi A., Tombal B. Omoumi P., Vande Berg B., Lecouvet F.E, *Radiology*. 2015 Apr; 275(1): 155–66. [43] With the permission of RSNA.

The 3D sequence is superior to the 2D concerning the quality of image (signal-to-noise and contrast-to-noise ratios). On 3D T1 reading, significantly more (p < 0.05) patients with node metastasis are detected than on 2D T1 with or without PDFS (observer 1 : area under the curve (AUC) = 1.00 for 3DT1, 0.81 for 2DT1 + DPFS imaging, and 0.73 for 2DT 1; observer 2: AUC = 1.00 for 3DT1, 0.81 2DT1+PDFS, and 0.81 for 2DT1) (Figure 5). No significant difference was observed between sequences for detecting patients with bone metastases (*P* ≥ 0.317 for all) (Figure 6). Concerning the number of metastatic lesions detected by the observers, whole-body 3DT1 had a significantly higher sensitivity (p < 0.05) than 2DT 1 and 2DT1+PDFS for detecting adenopathies (observer 1: sensitivity = 100% for 3DT1, 44% for 2DT1+PDFS , and 33% for 2DT1; observer 2: sensitivity = 92% for 3DT 1, 39% for 2DT1+PDFS, and 39% for 2DT 1), while whole-body 2DT1 had a significantly lower sensitivity (p < 0.05) than 3DT1 and 2DT1+PDFS for detecting bone metastasis (observer 1: sensitivity = 98% for 3DT1, 83% for 2DT1+PDFS, and 63% for 2DT1; observer 2: sensitivity = 100% for 3DT1, 85% for 2DT1+PDFS, and 68% for 2DT1).

Inter-observer agreement with the 3DT1 sequence was higher than that with 2DT1 for the detection of node metastasis on a per-patient and a per-lesion basis. Only limited discrepancies were noted between observers with 3DT1 in the detection of LNs, which may be difficult on 2DT1. The agreement was also very good for detecting BM.

The sagittal plane can potentially replace the additional spine sequences and the axial plane can be used for the detection of adenopathies and the study of ‘difficult’ regions as the posterior elements of the spine and the rib cage.

### MRI protocol for comprehensive prostate cancer staging

**Figure 7:**
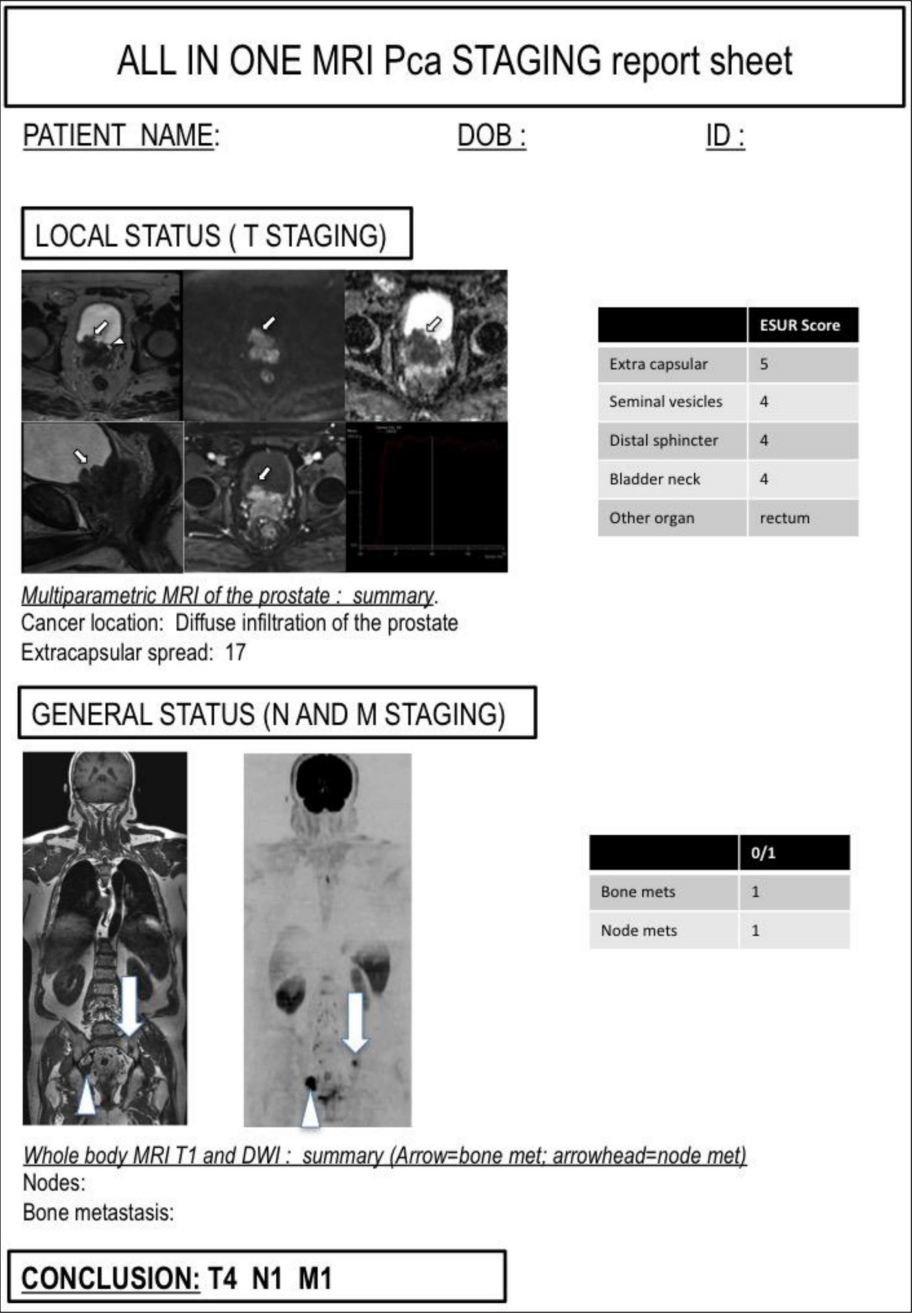
An example of an ‘all-in-one’ sheet, for the clinician with the summary of local, nodal and bone status of the patient.

After demonstrating that our 3D T1 anatomic sequence is superior to the combination of 2D sequences for the detection of prostate cancer metastasis we used it to build a new WB-MRI protocol for the simultaneous T, N, and M staging of PCa (Figure 7).

It has been demonstrated that in most PCa patients, the usual sites of metastases are the pelvic LNs and/or the hematopoietic red marrow of the axial skeleton [44,45,46]. Visceral metastases are rare and usually appear in later stages of the disease [45].

**Figure 8:**
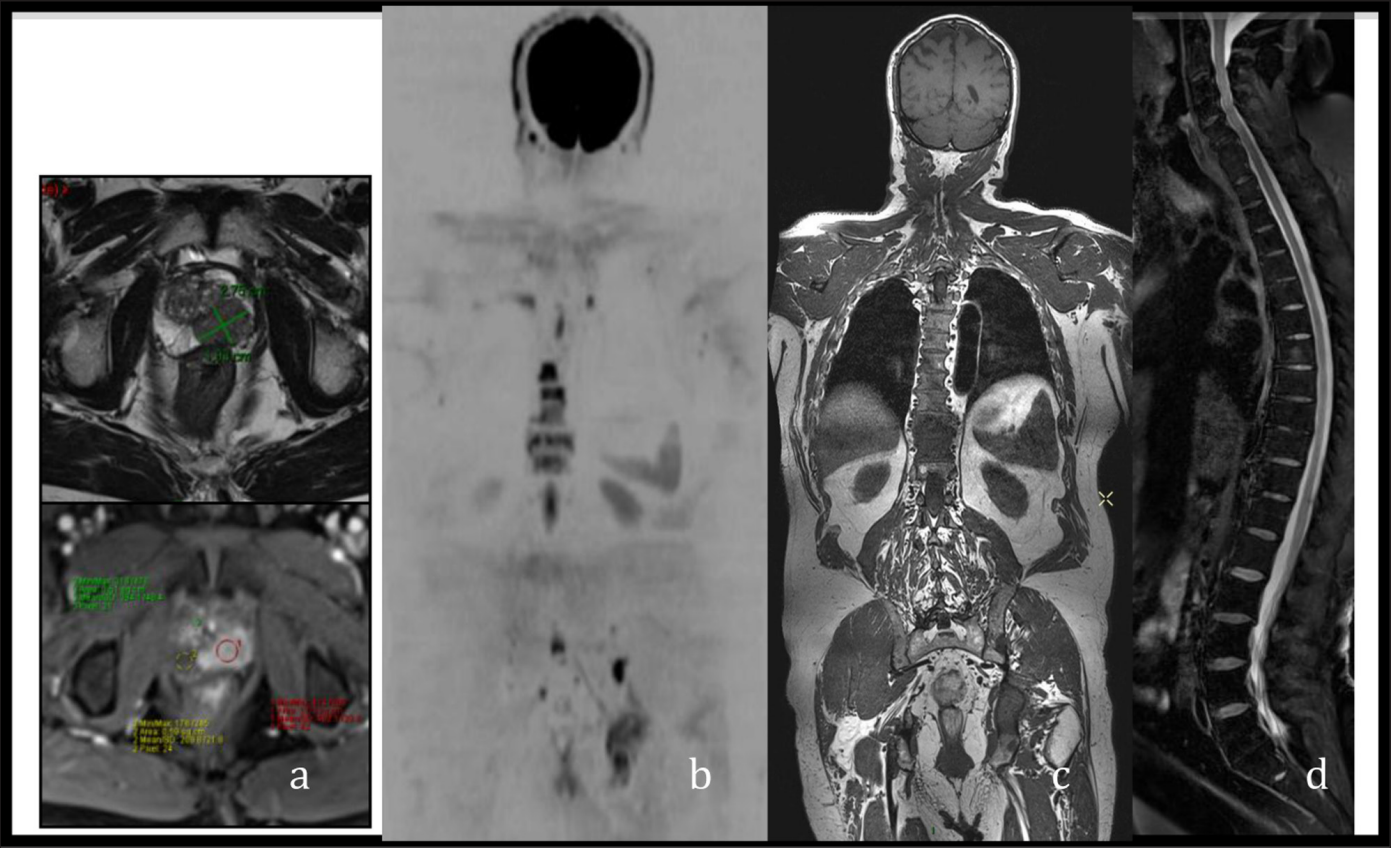
‘All-in-one’ MRI protocol for the concurrent TNM staging of PCa **(a)** mp-MRI of the prostate for the T staging **(b, c)** and **(d)** 3D T1 sequence, DWI and PDFS of the spine for the N and M staging.

**Figure 9:**
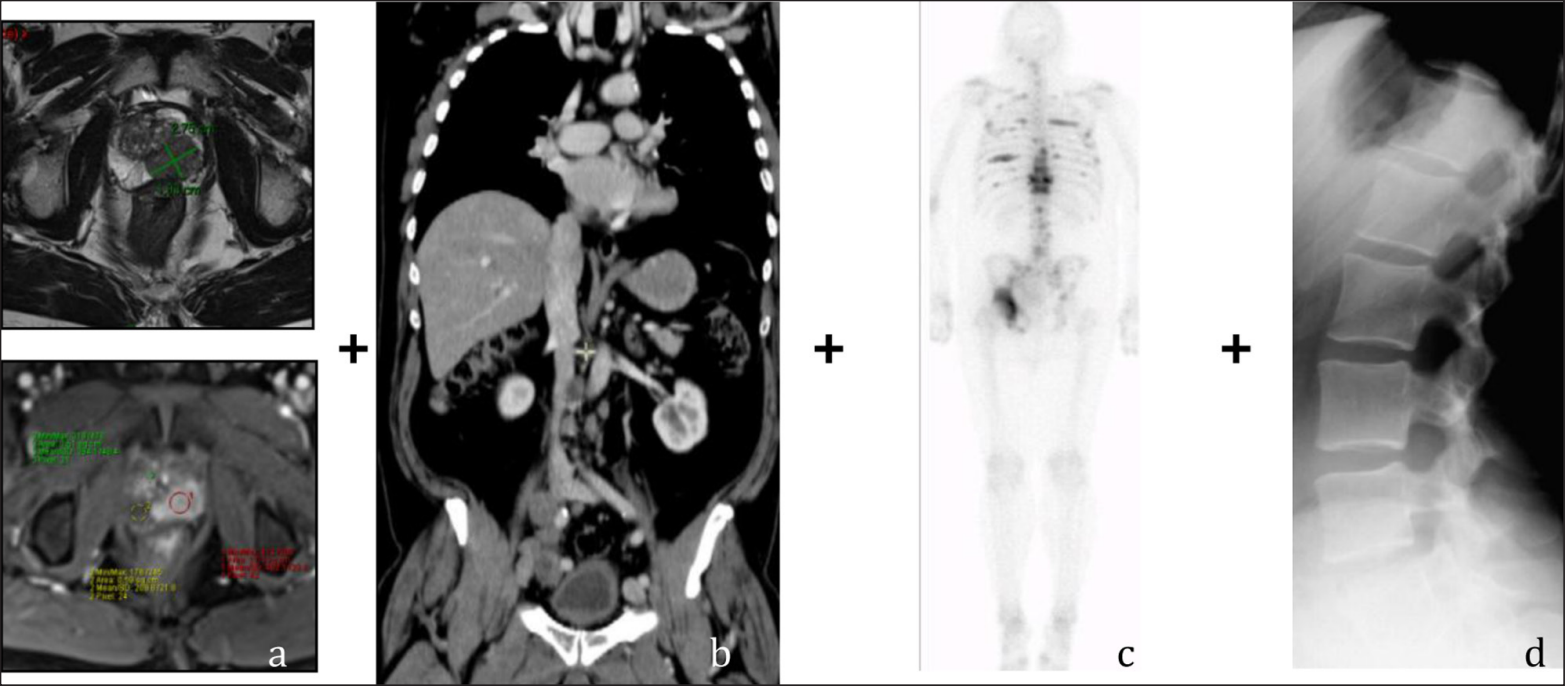
The multiparametric protocol **(a)** mp-MRI for the T staging **(b)** Thoraco-abdominal CT for the N staging **(c)** BS **(d)** Targeted X-Rays for the M-Staging.

Our goal was to assess the feasibility and value of an ‘all-in-one’ TNM staging protocol combining mp-MRI of the prostate for the local (T staging) with a WB-MRI protocol for the detection of node (N staging) and bone metastasis (M staging) (Figure 8). This new protocol could potentially replace the multiparametric protocol used until today: a mp-MRI for the local staging, a BS +/– targeted X-rays for the M staging and a Thoraco-abdominal CT for the N staging (Figure 9).

When we compare WB-MRI and routine work-up (combination of BS ± TXR and CT) for assessing global metastatic status (N and M Staging) of a PCa patient, WB-MRI is significantly superior for the detection of bone and node metastasis of PCa. For the detection of bone or node metastasis, sensitivities of BS ± TXR combined with CT and of WB-MRI were 85% and 100%, respectively, and specificities were 88% and 100%, respectively.

## Conclusions

WB-MRI is superior to BS for BM detection, independently of the primary cancer and the addition of DWI improves the sensitivity. Results are less clear-cut regarding the comparison between WB-MRI and PET-CT. WB-MRI appears to be superior in lesion-based analysis when PET-CT exceeds WB-MRI in patient-based analysis. Undeniably, current cancer staging cannot be based on one sole technique but it will most likely be shared between WB-MRI and PET according to the primary cancer. In PCa, in patient-based analysis, WB-MRI+DWI is more accurate than FDG PET-CT and BS.

We constructed a 3D T1 whole body sequence for the bone and node staging of high-risk PCa patients. We optimized the quality of image in comparison to the standard two-dimensional sequence and the duration of this new sequence was shortened/minimized down to 18 minutes. We introduced this sequence in clinical practice and compared it with current MRI anatomic protocol, which includes 2D T1 WB-MRI, with or without sagittal fat suppression sequence of the spine. Our results demonstrated that 3DT1 is feasible and provides better SNR and CNR compared with 2D sequences, with a diagnostic performance that is as good or better than the sum of 2D sequences for the detection of bone metastases and better for the detection of LNM.

Finally we developed a single step ‘all-in-one’ protocol for the staging of high-risk PCa patients consisting of a WB-MRI anatomic sequence including diffusion weighted imaging (DWI) and prostate mp-MRI. This new protocol was compared to the routine bone and node workup [BS ± targeted XRays if necessary (BS ± TXR) and contrast CT]. Our results demonstrated that TNM staging of PCa is feasible in less than one hour in an MRI session combining mp-MRI and WB-MRI, outperforming the routine screening for discriminating patients with or without metastasis.
